# Supplementary material for: Primed bovine embryonic stem cell lines can be derived at diverse stages of blastocyst development with similar efficiency and molecular characteristics
Source: Biol Open. 2025 Mar 7;14(3):BIO061819. doi: 10.1242/bio.061819 (PMC11911636; doi:10.1242/bio.061819)
Supplement: Supplementary information [file biolopen-14-061819-s1.pdf]

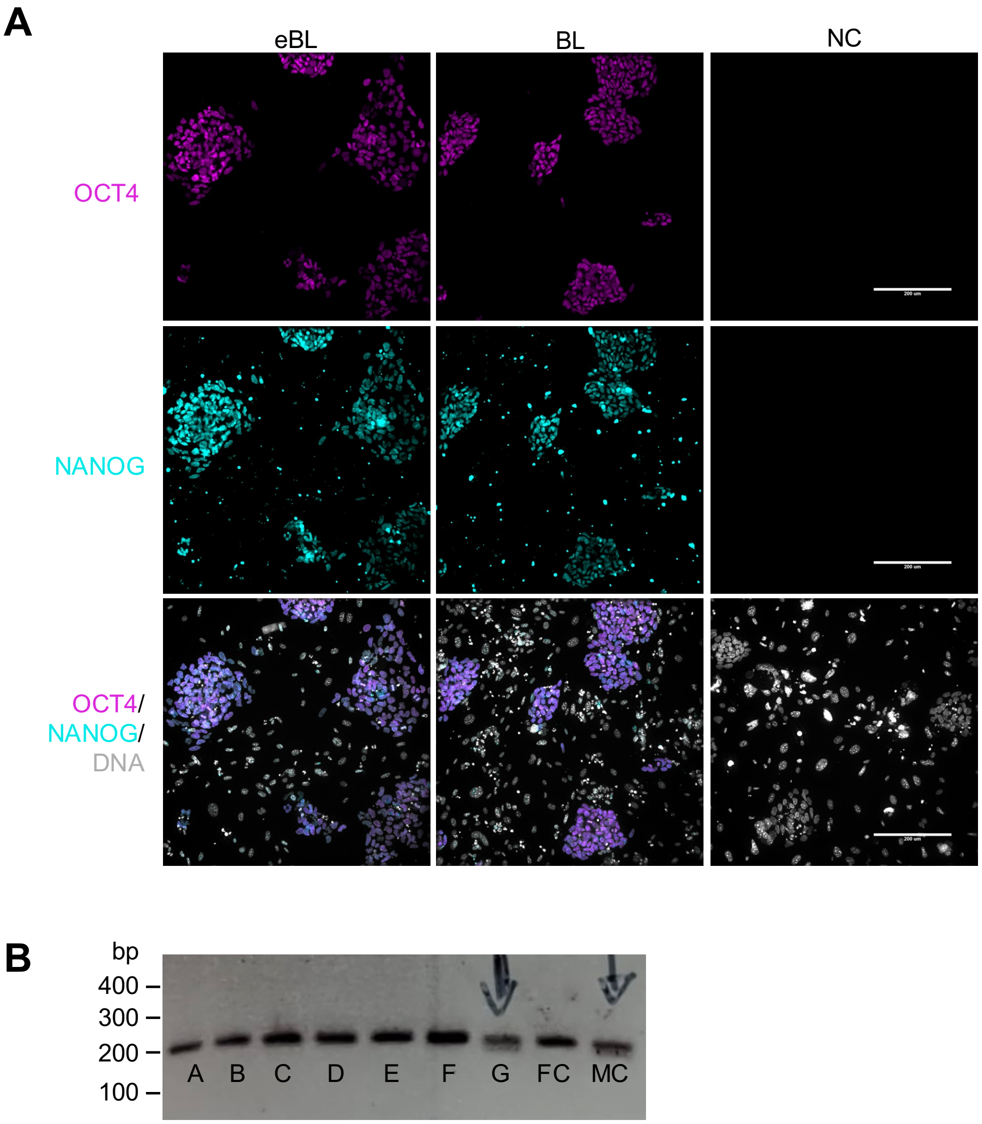

Figure S1. Additional characterization of bovine ESC lines from this study. (A) Representative immunofluorescence images of bESCs derived from early (eBL) and full (BL) blastocysts on MEF feeders at passage 16. Magenta indicates OCT4; cyan indicates NANOG; gray indicates DNA. NC is negative control, with no primary antibody added. Scale bar, 200 µm. **(B)** Sexing results of bESC lines visualized by agarose gel electrophoresis. A-G represent tested cell lines from this experiment. MC is male control, FC is female control. Arrows indicate samples determined as male, based on two bands.

Table S1. Antibodies used for immunolocalization analysis. Primary antibodies were reconstituted to the concentration specified by manufacturer instructions.

**Primary antibodies**	**Manufacturer**	**Catalog number**	**Dilution**
Goat anti-OCT3/4	R&D Systems	AF1759	1:100
Rabbit anti-SOX2	BioGenex	NU833-5UC	1:300
Rabbit anti-NANOG	Invitrogen	500-P236	1:200
Rabbit anti-H3K27me3	Millipore	ABE44	1:500
Mouse anti-H3K9me2	Abcam	AB1220	1:500
**Secondary antibodies**	**Manufacturer**	**Catalog number**	**Dilution**
Donkey anti-goat AlexaFluor 568	Invitrogen	A11057	1:500
Donkey anti-rabbit AlexaFluor 488	Invitrogen	A21206	1:500
Donkey anti-mouse AlexaFluor 546	Invitrogen	A10036	1:500
**Nuclear dye**	**Manufacturer**	**Catalog number**	**Dilution**
Hoechst 33342	BD Biosciences	BDB561908	1:1000

Table S2. Primer sequences for genes analyzed by polymerase chain reaction. Tm = melt temperature, F = forward and R = reverse primer sequences. X and Y indicate female and male sex chromosomes, respectively.

Target		Sequence	Product size (bp)
DDX3	F	AGGAAGCCAGGAAAGTAA	X: 208<br>Y: 184
	R	CATCCACGTTCTAAGTCTC	
OCT4	F	AACGAGAATCTGCAGGAGATATG	87
	R	TCTCACTCGGTTCTCGATACT	
SOX2	F	CATTAACGGCACACTGCCCC	76
	R	TGAAAATGTCTCCCCCGCCC	
NANOG	F	CAGCTACAAGCAGGTGAAGA	84
	R	CTATTCCTCGGCCAGTTGTT	
XIST	F	TTTACCCAACGGGGTCATGG	139
	R	AGGGGGTACACAGCAGGTAT	
PAX6	F	CCCTGGAGAAAGAGTTTGAGAG	126
	R	TCCATTTGGCCCTTCGATTAG	
H2A.2	F	GAGGAGCTGAACAAGCTGTTG	104
	R	TTGTGGTGGCTCTCAGTCTTC	

Table S3. Gene ontology analysis of enriched biological processes of bovine ESCs in various conditions. Enriched pathways based on differential gene expression of bovine ESCs cultured in N2B27 base (NBFR) vs. mTeSR-E6 base (MTFR) on top and bovine ESCs cultured in primed medium (NBFR) vs. expanded potential medium (EPSCM) on bottom.

**MTFR vs. NBFR**	p.geomean	stat.mean	p.val	q.val	set.size	exp1
GO:1902531 regulation of intracellular signal transduction	0.0018	2.9294	0.0018	0.6083	207	0.0018
GO:0010647 positive regulation of cell communication	0.0019	2.9158	0.0019	0.6083	187	0.0019
GO:0023056 positive regulation of signaling	0.0020	2.8908	0.0020	0.6083	188	0.0020
GO:0009967 positive regulation of signal transduction	0.0026	2.8179	0.0026	0.6083	171	0.0026
GO:0048583 regulation of response to stimulus	0.0029	2.7647	0.0029	0.6083	448	0.0029
GO:0010646 regulation of cell communication	0.0032	2.7363	0.0032	0.6083	372	0.0032
GO:0009966 regulation of signal transduction	0.0033	2.7207	0.0033	0.6083	333	0.0033
GO:0023051 regulation of signaling	0.0034	2.7098	0.0034	0.6083	373	0.0034
GO:1902533 positive regulation of intracellular signal transduction	0.0041	2.6694	0.0041	0.6083	116	0.0041
GO:0048513 animal organ development	0.0045	2.6229	0.0045	0.6083	271	0.0045
GO:0035556 intracellular signal transduction	0.0049	2.5903	0.0049	0.6083	299	0.0049
GO:0008285 negative regulation of cell population proliferation	0.0056	2.5772	0.0056	0.6083	74	0.0056
GO:0061061 muscle structure development	0.0066	2.5198	0.0066	0.6083	65	0.0066
GO:0048584 positive regulation of response to stimulus	0.0071	2.4611	0.0071	0.6083	252	0.0071
GO:0008283 cell population proliferation	0.0101	2.3283	0.0101	0.6083	315	0.0101
**NBFR vs. MTFR**	p.geomean	stat.mean	p.val	q.val	set.size	exp1
GO:0044272 sulfur compound biosynthetic process	0.0117	-2.3790	0.0117	0.9968	19	0.0117
GO:1901135 carbohydrate derivative metabolic process	0.0135	-2.2234	0.0135	0.9968	127	0.0135
GO:1901137 carbohydrate derivative biosynthetic process	0.0185	-2.1066	0.0185	0.9968	72	0.0185
GO:0019693 ribose phosphate metabolic process	0.0313	-1.8827	0.0313	0.9968	53	0.0313
GO:0009101 glycoprotein biosynthetic process	0.0330	-1.8958	0.0330	0.9968	21	0.0330
GO:0009259 ribonucleotide metabolic process	0.0351	-1.8297	0.0351	0.9968	52	0.0351
GO:0033865 nucleoside bisphosphate metabolic process	0.0407	-1.8210	0.0407	0.9968	13	0.0407
GO:0033875 ribonucleoside bisphosphate metabolic process	0.0407	-1.8210	0.0407	0.9968	13	0.0407
GO:0034032 purine nucleoside bisphosphate metabolic process	0.0407	-1.8210	0.0407	0.9968	13	0.0407
GO:0032392 DNA geometric change	0.0422	-1.7763	0.0422	0.9968	21	0.0422

GO:0071103 DNA conformation change	0.0422	-1.7763	0.0422	0.9968	21	0.0422
GO:0009156 ribonucleoside monophosphate biosynthetic process	0.0456	-1.8318	0.0456	0.9968	10	0.0456
GO:0070085 glycosylation	0.0467	-1.7323	0.0467	0.9968	19	0.0467
GO:0006261 DNA-templated DNA replication	0.0468	-1.7173	0.0468	0.9968	26	0.0468
**EPSCM vs. NBFR**	p.geomean	stat.mean	p.val	q.val	set.size	exp1
GO:0022613 ribonucleoprotein complex biogenesis	0.0001	3.7140	0.0001	0.1959	85	0.0001
GO:0006412 translation	0.0006	3.2856	0.0006	0.2907	122	0.0006
GO:0042254 ribosome biogenesis	0.0006	3.3161	0.0006	0.2907	55	0.0006
GO:0043604 amide biosynthetic process	0.0009	3.1403	0.0009	0.3178	138	0.0009
GO:0043043 peptide biosynthetic process	0.0012	3.0584	0.0012	0.3204	127	0.0012
GO:0044085 cellular component biogenesis	0.0014	2.9958	0.0014	0.3204	354	0.0014
GO:0043933 protein-containing complex organization	0.0025	2.8144	0.0025	0.4401	261	0.0025
GO:0006518 peptide metabolic process	0.0027	2.8041	0.0027	0.4401	144	0.0027
GO:0065003 protein-containing complex assembly	0.0029	2.7730	0.0029	0.4401	191	0.0029
GO:0045861 negative regulation of proteolysis	0.0042	2.8298	0.0042	0.5643	18	0.0042
GO:0044271 cellular nitrogen compound biosynthetic process	0.0051	2.5754	0.0051	0.6278	420	0.0051
GO:0007005 mitochondrion organization	0.0062	2.5244	0.0062	0.6451	92	0.0062
GO:0008202 steroid metabolic process	0.0065	2.5875	0.0065	0.6451	24	0.0065
GO:0022900 electron transport chain	0.0067	2.5923	0.0067	0.6451	22	0.0067
GO:0032543 mitochondrial translation	0.0086	2.4859	0.0086	0.7169	25	0.0086
**NBFR vs. EPSCM**	p.geomean	stat.mean	p.val	q.val	set.size	exp1
GO:0001944 vasculature development	0.0237	-2.0101	0.0237	0.9843	47	0.0237
GO:0035295 tube development	0.0251	-1.9749	0.0251	0.9843	70	0.0251
GO:0001568 blood vessel development	0.0296	-1.9111	0.0296	0.9843	46	0.0296
GO:0035239 tube morphogenesis	0.0316	-1.8778	0.0316	0.9843	54	0.0316
GO:1904377 positive regulation of protein localization to cell periphery	0.0330	-1.9786	0.0330	0.9843	10	0.0330
GO:0072359 circulatory system development	0.0358	-1.8158	0.0358	0.9843	72	0.0358
GO:0007041 lysosomal transport	0.0380	-1.9357	0.0380	0.9843	10	0.0380
GO:0007254 JNK cascade	0.0420	-1.8096	0.0420	0.9843	12	0.0420
